# Evaluating the impact of *Trichoderma* biofertilizer and planting dates on mustard yield performance using the InfoCrop growth model

**DOI:** 10.1371/journal.pone.0285482

**Published:** 2023-05-10

**Authors:** Shams Shaila Islam, A.T.M. Masum Billah, Ahmed Khairul Hasan, Rashed Karim, Thanet Khomphet

**Affiliations:** 1 Faculty of Agriculture, Department of Agronomy, Hajee Mohammad Danesh Science and Technology University, Dinajpur, Bangladesh; 2 Faculty of Agriculture, Department of Agronomy, Bangladesh Agricultural University, Mymensingh, Bangladesh; 3 New Government College, Rajshahi, Bangladesh; 4 School of Agricultural Technology and Food Industry, Walailak University, Thasala, Nakhon Si Thammarat, Thailand; ICAR-Indian Institute of Soil Science, INDIA

## Abstract

A crop simulation model is adopted to calculate the potential yield in a certain location. The data sets generated in each scenario (2021–2022) were used to evaluate the InfoCrop model. A field experiment using a randomized complete block design was conducted at the Agronomy Department’s research field, Hajee Mohammad Danesh Science and Technology University. The following two factors: 1) factor A: sowing dates (Planting date 1: PD_1_ = 5^th^ November and Planting date 2: PD_2_ = 15^th^ November 2021) and 2) factor B: *Trichoderma* biofertilizers (T_1_ = control, T_2_ = 50% chemical fertilizer + 2,000 kg ha^-1^ *Trichoderma* biofertlizer, T_3_ = fully chemical fertilizer; and T_4_ = fully 3,000 kg ha^-1^ *Trichoderma* biofertilizer). Three BARI (Bangladesh Agricultural Research Institute) released varieties (V_1_ = BARI Sarisa-14, V_2_ = BARI Sarisa-16, and V_3_ = BARI Sarisa-17) used for the completion of the experiment. The *Trichoderma* biofertilizer and planting dates had a significant influence on yield and yield attributes of mustard. Results showed that plant height, leaf width, leaves per plant, pods per plant, harvest index, maturity date, and yield were significantly affected by *Trichoderma* biofertilizer treatments, two different conditions, and varieties. The regression analysis indicated a significant linear relationship between two different growing conditions especially for harvest index PD_2_>PD_1_ (0.88>0.83), grain yield (0.94>0.90), flowering date (0.95>0.91) and maturity date (0.95>0.90). It was found that the model significantly overestimated all the parameters with an acceptable error range (<15%) while growth and yield characteristics including flowering and maturity dates and yield were simulated and results were compared to observed data. BARI Sarisa 16 had the highest simulated yield of 2.5 t ha^-1^ and showed a high yielding variety among the used varieties in the experiment. As a result, it can be concluded that if the InfoCrop growth model is carefully calibrated, it will be an excellent tool for evaluating and identifying the best yielding variety.

## Introduction

Mustard is a crop that is extremely susceptible to changing climatic conditions and has a big impact on its productivity. In Bangladesh, mustard is grown to extract oil from the seeds, which is used in cooking. Due to insufficient output in comparison to the country’s demand for edible oil, a substantial amount of mustard seed must be imported from outside. Every year, around 3 lakh tons of mustard seeds are imported, whereas the country produces 1 lakh tons of oilseed. Currently, 0.24 million hectares of land are under cultivation, with an annual yield of 0.19 million MT [1]. Furthermore, unbalanced usage of chemical fertilizers is a typical issue that leads to mustard output loss due to depleting soil fertility. Farmers in Bangladesh grow mustard seeds with chemical fertilizers such as urea, triple superphosphate (TSP), and muriate of potash (MOP). Although they use these chemical fertilizers in the hope of increasing yield, they are unaware of the proper doses of application.

To increase crop production, a large number of chemical fertilizers combined with a limited percentage of biofertilizers are applied and have an impact on soil and humans [2]. As a result, it alters the concentrations of micronutrient shortages, which in turn affects the output of various crops [3]. Another factor is that the organic matter content of the country’s soils is poor, with the majority of them being below the threshold level (1.5%) and having gradually decreased by 5 to 36% between 1967–2018 [4]. Thus, biofertilizer management may be the most effective long-term strategy for preserving soil fertility and productivity [5].

*Trichoderma* as a biofertilizer has the ability to synthesize fungal antibiotics, parasitize some other fungi, and compete against dangerous pathogens, which are considered the reasons why it has favorable impacts on plant growth and development [6]. *Trichoderma* can be found in many ecosystems and some strains have the ability to inhibit plant pathogens, mainly in the soil or plant roots, through high antagonistic and mycoparasitic potential [7]. It increases root growth and hair production, resulting in more efficient usage of nitrogen, phosphorus, potassium, micronutrients, seedling vigor, and germination rate [8]. Thus, a combination of chemical fertilizer and *Trichoderma* biofertilizer is applied to crop fields for increased crop output [9].

Lone et al. [10] stated that crop simulation models are valuable tools for investigating the dynamic relationships among numerous factors that influence crop production, such as weather, crop management, and soil conditions. Because modeling techniques can be used to extend study outcomes from one season or area to another season, location, or management, they may reduce the costly and time-consuming field experiments [11]. InfoCrop growth simulation model is one of the user-friendly dynamic crop growth models. This model has the capability to estimate the potential yield, simulated yield, yield gaps, and assess the impacts of climatic fluctuation on climate change. It can simulate the crop growth processes *viz*. phenology, interaction among varieties, treatments, environment, management, yield forecast, and yield loss of the mustard crop [12]. Therefore, the main objective of this study was to find the effect of *Trichoderma* biofertilizer at different planting dates to improve mustard production and identify high-yielding mustard variety with appropriate application rates of *Trichoderma* biofertilizer.

## Materials and methods

### Research place and experimental design

The experiment was carried out during (December-June) 2021–2022 cropping season at the Agronomy Department’s research field of Hajee Mohammad Danesh Science and Technology University (HSTU), Bangladesh. The research area is at latitude of 25.13°N, longitude of 88.23°E, and elevations of 37.5 m. The experiment was carried out with two factors, *i*.*e*., two planting dates were considered as factor A and different rates of *Trichoderma* (*Trichoderma harzianum*) biofertilizer application as factor B. In the case of the planting date, two dates were fixed at an interval of 10 days (PD_1_ = 5^th^ November and PD_2_ = 15^th^ November 2021). On the other hand, four treatments were determined for the application of *Trichoderma* biofertilizer (T_1_ = control, T_2_ = 50% chemical fertilizer (CF) + 2,000 kg ha^-1^ *Trichoderma* biofertilizer, T_3_ = fully *i*.*e*., 100% CF, and T_4_ = fully 3,000 kg ha^-1^ *Trichoderma* biofertilizer. The varieties used in the experiment were V1 = BARI Sarisa-14, V2 = BARI Sarisa-16, and V3 = BARI Sarisa-17). The experiment was conducted as a split plot using a randomized complete block design as main plots with three replications. The varieties were arranged to the main plot and *Trichoderma* enriched biofertilizer to the sub-plot. The total number of treatments was twelve (3× 4) with three replications. There were 36 plots for each planting date out of a total of 72 plots and each plot was 4×2.5 m in size. The recommended dosage of fertilizers per hectare were applied for mustard as 40 kg K_2_O, 60 kg P_2_O_5_, and 60–90 kg urea. According to the treatment schedule for fertilizer application, the treatments applied to the individual plots between the basal and flowering stages. Yield attributes collected for preparing InfoCrop growth model were plant height, leaves per plant, leaf width, pods per plant, harvest index, flowering date, maturity date, and finally yield.

### Statistical analysis

The analysis of variance (ANOVA) was performed and the relationship between *Trichoderma* biofertilizer treatments and varieties, different planting dates with yield attributes of mustard were analyzed by regression model using Statistix 10.1 and illustrated using Microsoft Excel. The mean comparison followed least significant difference (LSD) and reported at a 5% probability level. The Peason’s correlation coefficients were computed and visualized to determine the association among the yield attributes according to the “Corrplot” package of the R program. Weather graphs done by Origin Pro 2021, version-9.8.200.

### Description of the InfiCrop growth model

The climate change’s impact and response on mustard in Bangladesh studied using the InfoCrop model. The following five input files were prepared to run the model:

#### 1. Daily weather data

Maximum and minimum temperatures, rainfall, precipitation, and solar radiation were recorded from the weather station, Dinajpur Meteorological Office, Bangladesh.

#### 2. Soil characteristics

Soil characteristics were collected at the depts of 0–10, 10–30, 30–50, and 50–80 cm before started the research experiment. Soil samples were classified the types and analysed the properties included sand-silt-clay ratio, organic matter and carbon, total nitrogen (total N), total phosphorus (total P), available potassium (available K), available sulphur (available S), available zinc (available Zn), available boron (available B), field capacity, cation exchange capacity (CEC), electrical conductivity (EC), and soil pH.

#### 3. Planting management

Planting management such as planting density, row and date, field irrigation, weed control, sowing depth, fertilizer type, and insecticide application were recorded for calibrating the models.

#### 4. Plant data

The plant data were recorded included germination, flowering, and harvesting dates, plant population, plant height, tillers number, leaf width, leaves per plant, 1,000 grain weight, harvest index, pods per plant, fresh weight, dry weight, biological yield, economical yield, and yield per variety (grain yield per area).

#### 5. Genetic cultivar coefficient file

The parameters included Time-period or basic vegetative phase expressed as P1, critical photoperiod = P2O, photoperiodism coefficients = P2R, pods filling duration coefficient = P5, pods number coefficient = G1, single pod weight = G2, 1,000 pod weight coefficients = G3, and temperature tolerant coefficient = G4 were employed to calculate the genetic coefficients in the InfoCrop growth model presented in (Table 1).

**Table 1:** Genetic cultivar coefficients for the InfoCrop growth model.

Stage	Description
P1	Timing vegetative phase of the plant (stated as growing degree days [GDD] in°C) start at the time of seedling emergence throughout the mustard plant is not receptive to modifications in photoperiod.
P2O	Critical photoperiod or else the elongated day length (in hours) at that stage the growth happens at a highest rate.
P2R	Extent to which physiological improvement leads to branching opening is deferred (exhibited as GDD) for each hour increase in photoperiod above P2O.
P5	Timing in GDD from starting of pod filling (3–4 days after flowering) to physiological maturation at 9°C (base temperature).
G1	Potential pods number coefficient as calculated from the branch number gm^-1^ of main branch dry weight at the flowering time.
G2	With ideal growing conditions i.e., non-limiting sunlight, irrigation, fertilizer also absence of pests and diseases, it is calculated.
G3	A higher branching bearing variety must have the branching coefficient greater than 1.0.
G4	Calculated as 1.0 for varieties that grow up in normal environments or seasons.

## Result and discussions

### Soil physico-chemical properties

The chemical and physical properties of soils were analysed at the Soil Resource Development Institute (SRDI), Dinajpur, Bangladesh. It indicated that soil was loamy textured. Results (Table 2) showed that the experimental research field was acidic soil with poor physical and chemical characteristics which contains average sand, silt, and clay at 42.11, 40.63, and 15.86%, respectively, and the field capacity of soil was 10.40%. Soil contains 0.16% of organic carbon, 0.24% of organic matter, 0.16% of total nitrogen, 15.10% of total phosphorus, 0.22% of available potassium, 15.61% of available sulphur, 0.48% of available zinc, 0.55% of available boron, 2.20 Meq 100g^-1^ of cation exchange capacity, 76.41 mS cm^-1^ of electrical conductivity, and pH in water at 6.00. Based on these findings, it was stated that appropriate soil parameter measurement required for reliable model predictions in acidic soil conditions.

**Table 2:** Soil physico-chemical analyses at different layers.

Parameters measured	Units	Layers (cm)				
		0–10	10–30	30–50	50–80	Average
Soil textural classes	-	Loam	Loam	Loam	Loam	Loam
Sand	%	41.00	42.00	43.20	42.25	42.11
Silt	%	42.20	42.30	35.00	43.00	40.63
Clay	%	12.20	15.40	22.40	13.45	15.86
Organic matter	%	0.22	0.25	0.28	0.20	0.24
Organic carbon	%	0.18	0.14	0.16	0.14	0.16
Total N	%	0.015	0.24	0.107	0.02	0.16
Total P	%	16.81	15.20	13.40	14.40	15.00
Available K	mg kg^-1^	0.25	0.17	0.12	0.35	0.22
Available S	mg kg^-1^	15.85	14.82	16.09	15.67	15.61
Available Zn	µg g^-1^	0.37	0.44	0.56	0.56	0.48
Available Boron	µg g^-1^	0.14	0.20	1.52	0.35	0.55
Field capacity	%	10.90	10.50	10.00	10.20	10.40
CEC	meq 100g^-1^	2.14	2.97	1.00	2.66	2.20
EC	mS cm^-1^	79.00	75.30	77.00	74.35	76.41
pH	-	6.00	6.10	5.70	6.00	6.00

### Weather condition

**Fig 1:**
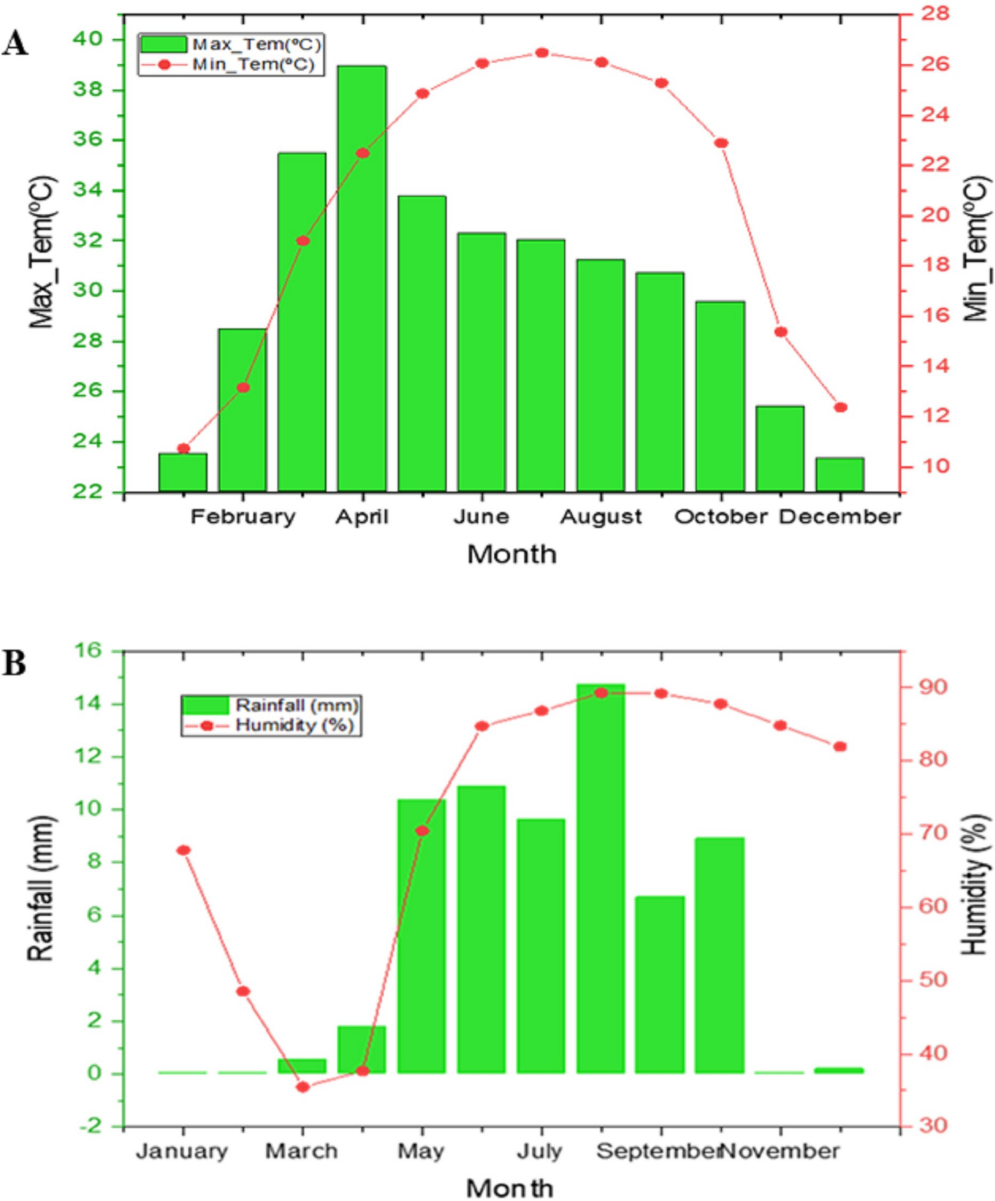
Weather conditions during the experimental year. A: Maximum and Minimum temperature and B: Cumulative rainfall and relative humidity.

Fig 1A and 1B showed that weather conditions had visible variations between the experimental months (2021–2022). Graphical representation showed that mean daily maximum and minimum temperatures ranged from 23 to 39°C and 12 to 26°C. However, the mean maximum and minimum temperatures were slightly different from planting to flowering and from flowering to maturity during the growing season. The highest and lowest maximum temperatures were observed in April (39°C) and December (22°C), respectively. The highest and lowest minimum temperature was observed in July (26°C) and December (10.5°C), respectively.

The highest rainfall was observed in September (1,400 mm), followed by June (1,100 mm) while there was no rainfall in November, January to March (0 mm) (Fig 1B). The rainfall distribution had a high variation during planting to flowering periods and flowering and physiological maturity. Relative humidity (RH) directly affects plant water uptake, which indirectly affects leaf growth, photosynthesis, pollination, disease incidence, and ultimately economic yield. The average relative humidity also supports the rainfall scenario and was 30–90% with highest and lowest RH were observed in September to October (90%) and March (30%), respectively. Hence, appropriate sunshine hours are particularly helpful during particular crop stages such as grain filling and maturity period in case sufficient water supply.

### Calculated genetic cultivar coefficients

The genetic cultivar coefficients of InfoCrop model were calibrated through observation time obtained from the field experiment at different treatments of *Trichoderma* biofertilizer levels. To examine the best agreement, the “trial and error” method was utilized for calibrating, for instance, root mean square error (RMSE) was used to determine the lowest differences between the simulated and measured yield by altering crop growth variables. The calibrated mustard coefficients provide better agreement between simulated and measured yields. Thermal time for different phases obtained from Table 3. Result showed that highest germination time (110), seedling emergence to flowering days (910) obtained from BARI Sarisa 14. Maximum specific leaf area (0.0024), potential growth rate (2.08), potential rooting depth rate (47), maximum number of pods (45,658,000), potential weight of a grain (6.50) obtained from BARI Sarisa 16. The highest flowering to maturity date in degree day (1,090) obtained from BARI Sarisa 17.

**Table 3:** Calculated genetic cultivar coefficient values for three mustard varieties.

Thermal time	Units	BARI Sarisa 14	BARI Sarisa 16	BARI Sarisa 17
Germination	Degree day	110	100	95
Seedling emergence to flowering	Degree day	910	880	890
Flowering to maturity	Degree day	1,070	1,020	1,090
Specific leaf area	Fraction	0.0022	0.0024	0.0023
Potential growth rate	Fraction	2.02	2.08	2.07
Potential rooting depth growth rate	mm d^-1^	43	47	46
Maximum number of pods	Pods per hectare	42,565,000	45,658,000	43,506,000
Potential weight of a grain	mg grain^-1^	4.80	6.50	6.20

### Analysis of variance result for crop performance

Two-factor ANOVA analysis was done using Rstudio, version-4.2.1 and the results support the InfoCrop growth model [13]. The statistical analysis result in Table 4. showed that all yield attributes exhibited highly significant differences among the three replications. Results revealed that control application of biofertilizers did not show significant effect but growth and yield of mustard increased significantly when *Trichoderma* was supplemented with NPK fertilizers [6]. The results also showed that only biofertilizer or *Trichoderma* application significantly increased mustard production and achieved the highest crop yield. However, the best results were obtained using only *Trichoderma*. The *Trichoderma* biofertilizer treatments and varieties also demonstrated highly significant differences. The most significant variations between varieties were found in their phenological parameters. The result indicated that plant height (311.6 cm), harvest index (106.79), leaf width (4.10 cm), leaves per plant (32.46 number), pods per plant (54.70 number), yield (4.59 t ha^-1^), and flowering date (0.67 days) were significantly affected by treatments (4 doses of *Trichoderma* biofertilizer), sowing dates (5 November and 15 November), and varieties (BARI Sarisa 14, BARI Sarisa 16, BARI Sarisa 17). The results of replication to replication showed non-significant or very few differences. Only the flowering date had no significant performance with the treatments and other parameters.

**Table 4:** F–values and significance obtained from the analysis of variance for yield contributing traits influenced by various *Trichoderma* biofertilization, two growing dates (PD_1_ and PD_2_), and variety.

Sources	df	Mean squares							
		PH	L_P	L_W	P_P	HI	GY	FD	MD
Treatment (T)	3	311.6**	32.46**	4.10**	54.70**	106.79*	4.59**	0.67**	1.85^ns^
Planting date (PD)	1	420.8**	288.00**	3.65**	1.67**	4.60^ns^	0.39*	0.001^ns^	0.10^ns^
Replication (R)	2	1444.9**	131.26^**^	0.042^ns^	86.22**	45.84^ns^	0.01^ns^	0.06^ns^	5.39^ns^
Variety (V)	2	1499.3**	5.7^ns^	0.07*	16.69^ns^	9.68^ns^	0.29**	8.72**	53.39**
T × V	6	5439.1 ^ns^	88.26^ns^	1.08*	3.05^ns^	42.88**	0.37**	38.06**	186.13**
V × PD	2	6.1**	8.29^ns^	0.03^ns^	8.01**	1.54**	0.01^ns^	0.010^ns^	0.10^ns^
T × PD	3	0.6**	38.19^ns^	0.05^ns^	25.87^ns^	58.10^ns^	0.03^ns^	0.010^ns^	0.10^ns^
V ×T × PD	6	4.0**	10.09**	0.02^ns^	8.16**	17.55**	0.04^ns^	0.010^ns^	0.10^ns^
Pooled error	46	113.7	16.18	0.02	11.03	29.99	0.06	1.65	2.32
CV (%)		14.73	23.85	8.43	25.99	21.45	15.56	3.89	1.84

**Note:** PH = Plant Height; MD = Maturity Date; FD = Flowering Date; GY = Grain Yield; P_P = Pods per Plant; HI = Harvest Index; L_P = Leaves per Plant; L_W = Leaves per Width; ns: non-significant; *^,^**:*p* < 0.05 and 0.01, respectively.

### Regression analysis for crop performance

#### 1. Phenology

The regression analysis for plant height indicated a very low significant linear relationship between two different growing dates. While leaves per plant, leaf width, grain yield, pods per plant, and flowering and maturity days were highly significantly affected by growing days. In addition, a very poor relationship for harvest index continued to increase with the increase in *Trichoderma* biofertilization. It was established that the crop duration was significantly shorter on the second than the first planting date, perhaps because of the local climatic condition.

**Fig 2:**
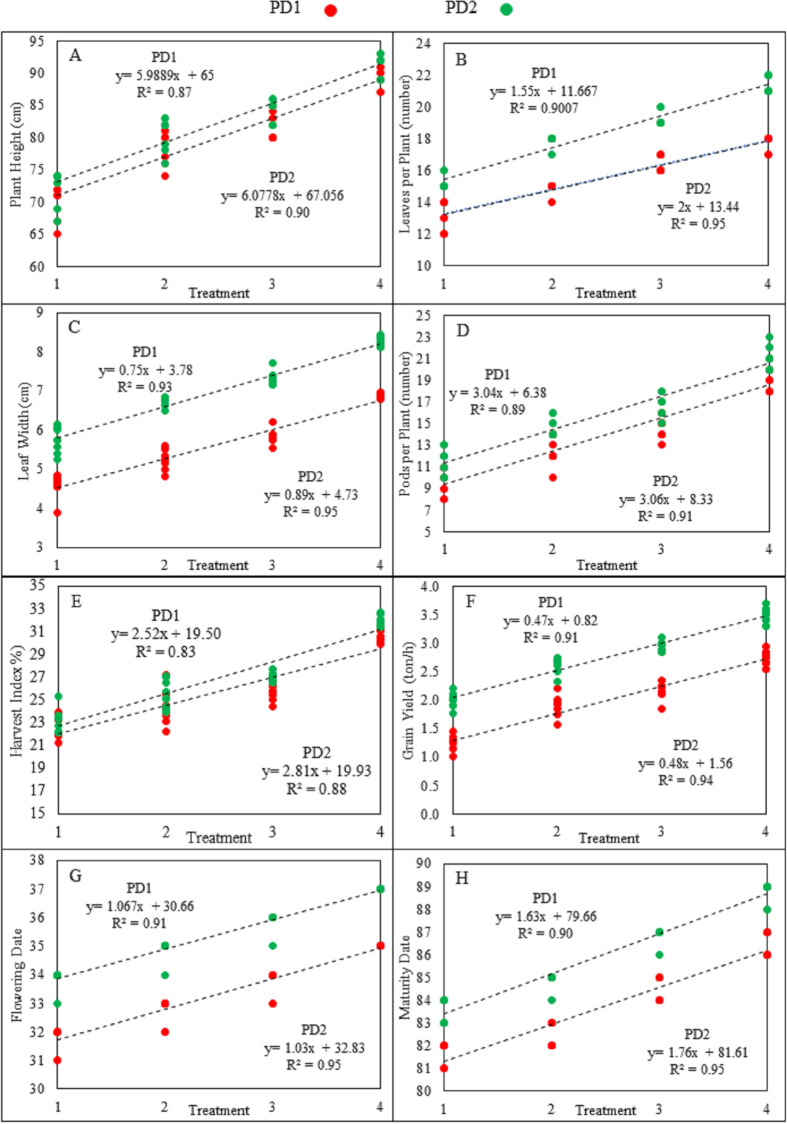
Linear regression relationships between *Trichoderma* biofertilizer treatments with different planting dates with Plant height (A), leaves per plant (B), leaf width (C), pods per plant (D), harvest index (E), grain yield (F), flowering days (G), and maturity days (H). *** Significant at *p* < 0.001.

*1*.*1 Plant height*: *Trichoderma* bio fertilization and different planting dates significantly affected plant height. Plant height increased with an increased fertilizer treatment, and maximum plant height was observed at T_4_ i.e., application of 3,000 kg ha^-1^ *Trichoderma* under both the planting date. An increase in plant height under the effect of biofertilizer addition ranged from (70–78) cm PD_1_, and (72–80) cm under PD_2_ planting date. Under the impact of planting date, maximum plant height was observed in PD_2_ at all biofertilizer treatments. Plant height increased by 72, 74, 78, and 80 cm for T1, T2, T3, and T4, respectively, under PD_2_ while 70, 72, 74, and 78 cm respectively increased under PD_1_ (Fig 2A). PD alone had a significant positive impact on plant height showed regression value PD_2_>PD_1_ (0.90>0.87).

*1*.*2 Leaves per plant*: Leaves per plant increased with an increased fertilizer treatment, and maximum was observed at T_4_ i.e., application of 3,000 kg ha^-1^ *Trichoderma* biofertilizer under both the planting date. An increase in leaves per plant under the effect of biofertilizer addition ranged from 12–13 PD_1_, and 15–18 with PD_2_ planting date. Under the impact of planting date, maximum leaves number was observed in PD_2_ at all biofertilizer treatments. Leaves per plant increased by 15, 15, 16, and 18 number for T1 to T4 treatments respectively, under PD_2_ while 12, 12, 13, and 12 respectively under PD_1_ (Fig 2B). Therefore, PD showed a significant positive impact on leaves per plant showed regression value PD_2_>PD_1_ (0.95>0.90).

*1*.*3 Leaf width*: Maximum leaf width was observed at T_4_ i.e., application of 3,000 kg ha^-1^ *Trichoderma* biofertilizer under PD_2_ planting date (6 cm). Result showed that leaf width was 4 cm for all the treatments with PD_1_, and 6–7 cm with PD_2_ planting date. Under the impact of planting date, maximum leaf width was observed in PD_2_ at all biofertilizer treatments (Fig 2C). Therefore, PD showed a significant positive impact on leaf width with regression value PD_2_>PD_1_ (0.95>0.93).

*1*.*4 Pods per plant*: Maximum pods per plant was observed at T_4_ i.e., application of 3,000 kg ha^-1^ *Trichoderma* biofertilizer under PD_2_ planting date (15). The result showed that pods per plant were 10 for all the treatments with PD_1_, and 12–15 with PD_2_ planting date. Under the impact of planting date, maximum pods per plant were observed in PD_2_ at all biofertilizer treatments (Fig 2D). Therefore, PD showed a significant positive impact on leaf width with regression value PD_2_>PD_1_ (0.91>0.89).

*1*.*5 Harvest index*: The harvest index had no visible difference between the planting dates. Harvest index 20–22% observed for all the treatments (Fig 2E). Therefore, harvest index showed regression value PD_2_>PD_1_ (0.88>0.83).

*1*.*6 Grain yield*: Grain yield increased with an increased biofertilizer treatment, and maximum was observed at T_4_ i.e., application of 3,000 kg ha^-1^ *Trichoderma* biofertilizer under both the planting dates. An increased grain yield under the effect of biofertilizer addition ranged from 1–1.5 t ha^-1^ with PD_1_, and 2–3 t ha^-1^ with PD_2_ planting date. Under the impact of planting date, maximum grain yield was observed in PD_2_ at all biofertilizer treatments. Grain yield increased by 1, 1.1, 1.2, and 1.5 t ha^-1^ for T_1_ to T_4_ treatments respectively, under PD_1_ while 2, 2.1, 2.3, and 2.5 t ha^-1^ respectively under PD_2_ (Fig 2F). Therefore, PD showed a significant positive impact on grain yield regression value PD_2_>PD_1_ (0.94>0.90).

*1*.*7 Flowering date and Maturity date*: The flowering date and maturity date had no visible difference from the planting dates. The flowering date showed 32 days for all the treatments with PD_1_ while 34 days with PD_2_ (Fig 2G). Therefore, flowering date had a regression value PD_2_>PD_1_ (0.95>0.91). Maturity date differ 81 days with PD_1_ and 83 days with PD_2_ (0.95>0.90) regression values (Fig 2H).

#### 2. Correlation coefficient

**Fig 3:**
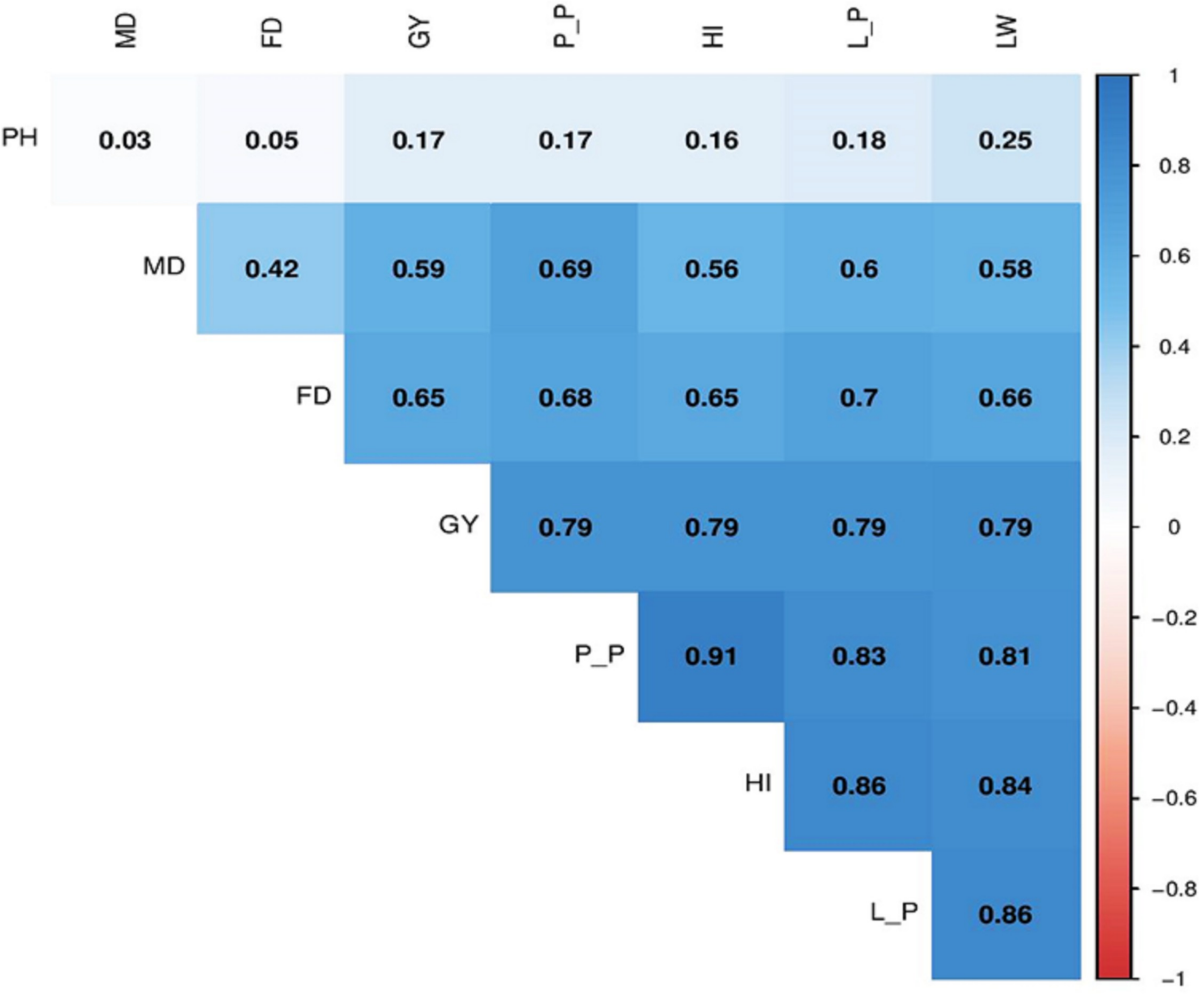
Plot of Pearson’s correlation analysis among the yield traits of mustard. Blue, dark blue and sky blue shaded indicated strong and positive correlation among yield attributes. Red color indicates negative correlation. The color intensity is proportional to the computed coefficients. PH = Plant Height, MD = Maturity Date, FD = Flowering Date, GY = Grain Yield, P_P = Pods per Plant, HI = Harvest Index, L_P = Leaves per Plant, and L_W = Leaves per Width.

Pearson’s correlation analysis on mustard yield traits revealed a strong correlation among all agronomic traits using *Trichoderma* biofertilizer treatments and various sowing dates on different mustard varieties (Fig 3). Pods per plant were very highly correlated with harvest index (0.91), leaves per plant (0.83), and leave width (0.81). The harvest index had a very strong positive association with the number of leaves per plant (0.86), as well as leaf width (0.84). Leaves per plant also showed a very high positive correlation with leaf width (0.86). Grain yield showed a high positive relationship with harvest index, pods and leaves per plant, and leaf width at 0.79. Plant height was very poorly correlated with all yield traits, except for leaf width (0.25). Maturity and flowering days were strongly correlated with all yield attributes.

#### 3. Comparison between measured and simulated yield of mustard sown on different dates

The result derived from the mustard InfoCrop model analysis indicates that compared to the simulated yield, the measured yield of BARI Sarisa 16 and BARI Sarisa 17 was higher among the varieties tested. Moreover, the model results also show that between BARI Sarisa 16 and BARI Sarisa 17, BARI Sarisa 16 is the more high-yielding variety. The model showed the average percent deviation and percent error for the mustard yield at various growing dates. The percentage deviation and percentage error values of the model derived from the late sowing date show that BARI mustard 16 is a high-yielding variety as compared to BARI mustard 14 and Sarisa 17 (Table 5). This suggests that the late sowing date was good for BARI mustard 16. It is underestimated on 5^th^ November (1.20 and 2.00) and 15^th^ November sown crop (0.98 and 0.99) and variety of BARI Sarisa 14 (0.95 and 4.00). There is a reduction in yield due to abnormal weather conditions leading to a greater loss in yield. The percent error showed for 4.00, 0.98, and 2.55 (BARI Sarisa 14, BARI Sarisa 16, and BARI Sarisa 17) and the average percent error for yield was found 1.8 (BARI Sarisa 17), 2.30 (BARI Sarisa 16) and 1.4 (BARI Sarisa 14), respectively. This showed that the evaluation of the model for yield revealed that the yield simulation found good with an acceptable level for mustard [14].

**Table 5:** Comparison between measured and simulated grain yield of mustard varieties sown on different dates.

Treatment	Grain yield (t ha^-1^)	
	Measured	Simulated
5^th^ November	1.2	1.3
15^th^ November	2.3	2.6
BARI Sarisa 14	1.4	1.6
BARI Sarisa 16	2.3	2.5
BARI Sarisa 17	1.8	2.0

#### 4. Model evaluation results represented by a 1:1 graph with an RMSE value

**Fig 4:**
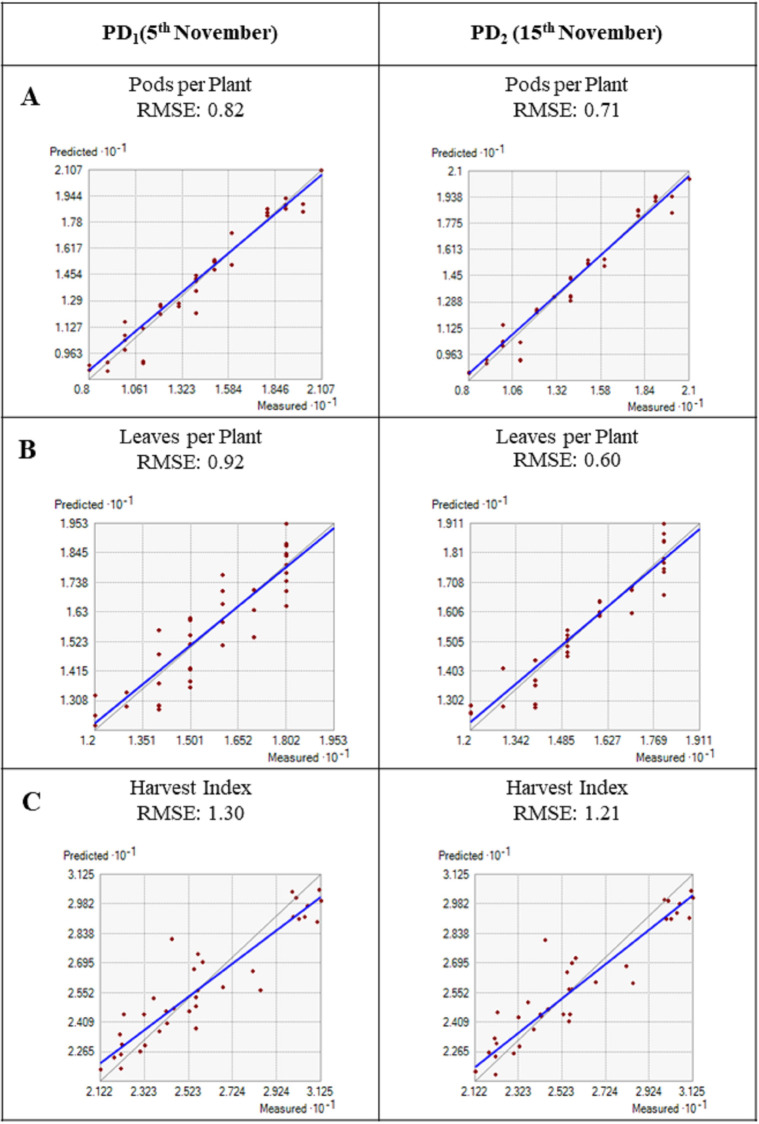
1: 1 graph showing the relationship between simulated and measured data. A: Pods per plant, B: Leaves per plant, and C: Harvest index.

**Fig 5:**
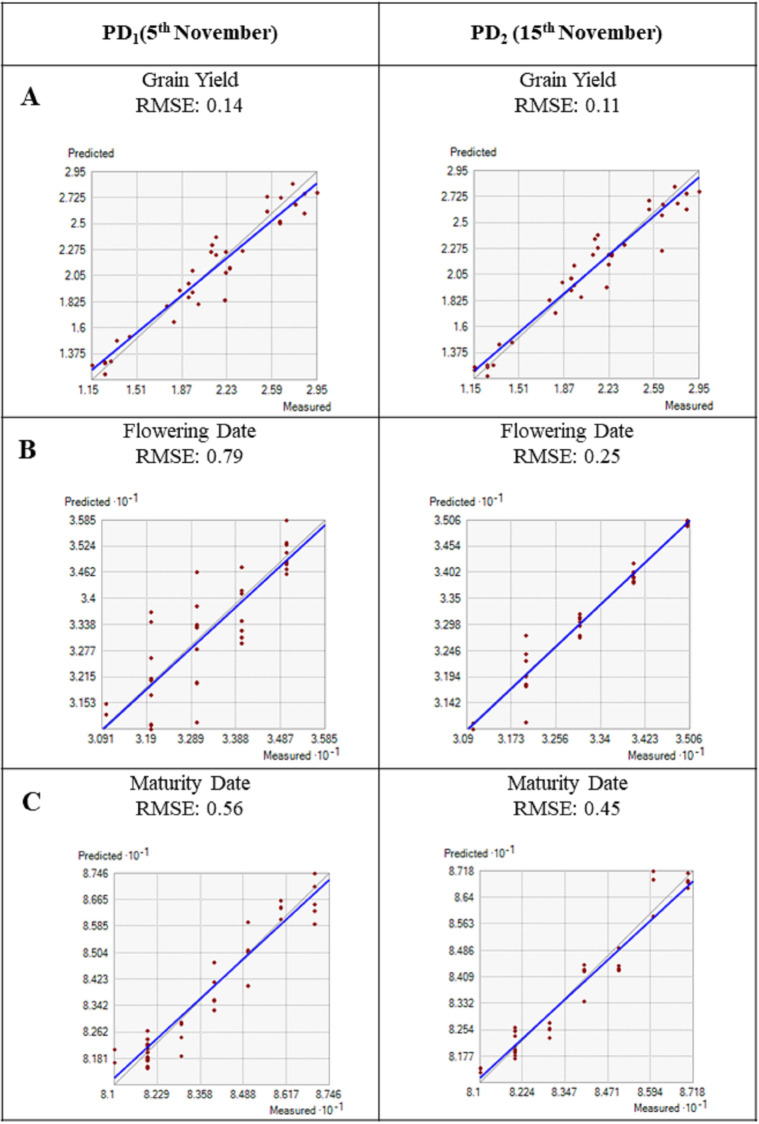
1: 1 graph showing the relationship between simulated and measured data. A: Grain yield, B: Flowering date, and C: Maturity date.

Model evaluation is an important phase in adjusting simulation results to ensure crop growth and yield parameters. To evaluate model performance, according to Hai-long et al. [15], five statistics were performed for estimating linear relationships including RMSE, normalized RMSE (n-RMSE), forecasting efficiency (EF), index of agreement (d), and coefficient of determination (R^2^). In this study, the n-RMSEs were classified as “good”, “moderate”, and “poor” agreement by ≤15, 15–30, and ≥30%, respectively. The RMSEs between measured and simulated values were classified as “excellent”, “good”, “moderate”, and “poor” when d>0.9, 0.8≤d<0.9, 0.7≤d<0.8, and d<0.7, respectively. Simulated pods per plant, leaves per plant, harvest index, grain yield, flowering, and maturity days were calculated by a 1:1 graph (Figs 4 and 5). However, the simulated grain yield was slightly higher than the observed data (Table 5) with the two different growing dates and showed superior performance for 15^th^ November i.e., PD_2_ had a strong agreement between simulated and measured values for yield attributes. PD_1_ (5^th^ November) showed poorer performance and had high RMSE values for flowering days (RMSE = 0.79), maturity days (RMSE = 0.56), leaves per plant (RMSE = 0.92), pods per plant (RMSE = 0.82), harvest index (1.30), and grain yield (RMSE = 0.14). PD_2_ showed best performing with flowering days (RMSE = 0.25), maturity days (RMSE = 0.45), leaves per plant (RMSE = 0.60), pods per plant (RMSE = 0.60). Similarly in the present study, mustard yield simulation was better with PD_2_ for grain yield (RMSE = 0.11). It is suggested that the InfoCrop growth model is more sensitive to PD_2_ (15^th^ November) than PD_1_ (5^th^ November) to real crop growth [15,16].

## Conclusion

The InfoCrop growth model was applied for the mustard with an application of *Trichoderma* biofertilizer under different planting dates to identify the high yielding mustard variety. The model included the HSTU soil, measured and simulated mustard yield, pods per plant, harvest index, and maturity days, and the results showed good agreement with various planting dates. The model proposed that the date 15^th^ November is optimal for maximum crop yield. The impact of *Trichoderma* biofertilizer application and sowing date on BARI Sarisa 14, BARI Sarisa 16, and BARI Sarisa 17 mustard experiments and analysis using the InfoCrop growth model revealed that the late sowing date (15^th^ November) and 3,000 kg *Trichoderma* biofertilizer application were the most effective for BARI Sarisa 16. The analysis of the model proved that the BARI Sarisa 16 to be the best variety among the varieties studied. In this case, the earlier sowing date (5^th^ November) combined with the application of 2,500 kg *Trichoderma* biofertilizer, and fully chemical fertilizer proved less acceptable. According to the findings of this research, different treatments and sowing dates have a significant impact on mustard production, and it is concluded that the response of the mustard crop to *Trichoderma* biofertilizer and planting date was not similar. Despite its limitations, this research can be regarded as a model study evaluating the impact of *Trichoderma* biofertilizer and planting dates on Mustard yield performance using the InfoCrop growth model.

## Supporting information

S1 Data (XLSX)

S1 File (DOC)

S2 File (DOC)

S3 File (DOC)

S4 File (DOC)

S5 File (TXT)
